# Bioprospection and Selection of Peptides by Phage Display as Novel Epitope-Based Diagnostic Probes for Serological Detection of HTLV-1 and Use in Future Vaccines

**DOI:** 10.3389/fmed.2022.884738

**Published:** 2022-06-09

**Authors:** Luiz Fernando Almeida Machado, Luiz Ricardo Goulart Filho, Fabiana Almeida Araújo Santos, Leonardo Quintão Siravenha, Andrea Nazaré Monteiro Rangel Silva, Maria Alice Freitas Queiroz, Antonio Carlos Rosário Vallinoto, Marluísa Oliveira Guimarães Ishak, Ricardo Ishak

**Affiliations:** ^1^Biology of Infectious and Parasitic Agents Post-Graduate Program, Federal University of Pará, Belém, Brazil; ^2^Virology Laboratory, Institute of Biological Sciences, Federal University of Pará, Belem, Brazil; ^3^Laboratory of Nanobiotechnology, Institute of Biotechnology, Federal University of Uberlandia, Uberlandia, Brazil

**Keywords:** HTLV-1, phage display, immunoglobulin G, serology, bioprospection

## Abstract

Human T-lymphotropic virus 1 (HTLV-1) is endemic worldwide and the infection results in severe diseases, including Adult T-cell Leukemia (ATL) and HTLV-1 associated myelopathy (HAM). There are some limitations of employing the present commercial serological assays for both diagnostic and epidemiological purposes in different geographical areas of the Brazil, such as the Amazon Region. Currently, methods for diagnosis are usually expensive to adapt for routine use. The aim of this work was to identify and characterize specific ligands to IgG that mimic HTLV-1 epitopes through the Phage Display technique, which could be used for diagnosis and as future vaccine candidates. Initially, IgG from 10 patients with HTLV-1 and 20 negative controls were covalently coupled to protein G-magnetic beads. After biopanning, genetic sequencing, bioinformatics analysis and Phage-ELISA were performed. The technique allowed the identification of 4 clones with HTLV-1 mimetic peptides, three aligned with gp46, A6 (SPYW), B6 (SQLP) and D7 (PLIL), and one with the protease and Tax, A8 (SPPR). Clones A6 and B6 showed higher values of accessibility, antigenicity and hydrophilicity. The reactivity of the clones evaluated by the Receiver Operating Characteristic (ROC) curve showed that the B6 clone had the highest Area Under Curve (0.83) and sensitivity and specificity values (both were 77.27 %; *p* < 0.001). The study showed that the Phage Display technique is effective for the identification of HTLV-1-related peptides. Clone B6 indicated to be a good marker for bioprospecting diagnostic test for HTLV-1 infection and could be used as a possible vaccine candidate for future studies.

## Introduction

Infection with human T-lymphotropic virus 1 (HTLV-1) can cause diseases such as adult T-cell leukemia/lymphoma (ATL) and HTLV-1-associated myelopathy (HAM) (1, 2). It is estimated that 5 to 10 million people are infected worldwide (3), but since most people are asymptomatic, the infection is neglected (4).

In Brazil, the diagnosis of HTLV infection presents great difficulties, since the tests for the virus are not fully available in the basic health care for all people. The test is requested for specific groups or situations, such as individuals with clinical manifestations compatible with diseases associated with HTLV-1, the differential diagnosis of myelopathies, during pregnancy and for blood donors. The diagnosis follows the algorithm of the Ministry of Health, which is still quite complex, expensive, and not available to the entire population (5, 6). As a general approach the laboratory diagnosis of HTLV infection includes screening procedures for the presence of antibodies including enzyme immunoassays or chemiluminescence assays, followed by complementary (confirmatory and discriminatory) tests (7). In Brazil, the national guidelines suggest the use of Western blot or the INNO LIA assays for those laboratories which do not have a molecular biology facility and other complex assays for those which are able to carry molecular complementary tests, including PCR and RT-PCR (5, 6). Complementary tests are expensive for most countries (3), but they are presently under study for their full inclusion in the Brazilian National Health System.

Although serological tests have been improved in recent years (8), the lack of standardization of tests with viral epitopes that are circulating in the country may lead to a high number of false-positive or indeterminate cases both for HTLV-2 (9) and to HTLV-1 (6). Antigenic differences among the molecular subtypes circulating are also a limitation factor which prevents the standardization of serological assays. However, the phage display assay is one of the approaches to select more specific peptides for improved serological tests.

The phage display technique consists of the presentation of peptides on the surface of filamentous bacteriophages. These peptide sequences can interact with a wide variety of target molecules, resulting in a physical link between the displayed peptide and the target molecule (10). The selection of exogenous peptides exposed on the surface of the phage takes into account its affinity for a target molecule by a process called biopanning, in which a phage library is presented to the target molecule (e.g., antibodies present in the serum of patients) and the phages with high affinity bind to the target while those with low affinity are removed. Phages with high affinity are recovered, amplified, and subjected to genetic sequencing to identify their peptide sequences, which are evaluated for their sensitivity and specificity at detecting the target molecule (11, 12).

Phage display is a low-cost method that yields relatively fast results, characteristics that have favored the use of the technique in the identification of peptides for use in different areas of health (12–15). The phage display technique has been used to identify peptides for the diagnosis of several human infectious diseases (16, 17) and possible vaccine candidates (18–20). This strategy is one possibility for identifying peptides that can be used in the construction of a human immunodeficiency virus (HIV-1) vaccine formulation (12). In this sense, this method can is a promising way to identify new, more specific targets that can be used in the diagnosis of HTLV-1 infection. The main objective of the present study was to search for new biomarkers mimicking HTLV-1 for the rapid diagnosis of infection as a portable, easy-to-use, sensitive instrument that can be used in difficult-to-access areas and clinics. We also hoped that these peptides could be used to induce the production of neutralizing antibodies for the manufacture of a future vaccine.

## Materials and Methods

### Characterization of the Samples

To select peptides to screen, serum samples were taken from people living with HTLV-1 from the Clinic of Infectious Diseases (Tropical Diseases) of the Center for Tropical Medicine, Institute of Health Sciences, Federal University of Pará (Universidade Federal do Pará), and an HTLV-negative control group taken from the general population of Belém (students, technicians, and professors of the Federal University of Pará) as well as blood donors from the state of Pará.

Forty individuals participated in the study; 10 individuals were diagnosed with HTLV-1 infection, and 20 individuals in the control group were seronegative for anti-HTLV-1/2 and anti-HIV-1 antibodies. To increase the specificity of the selected peptides, a subtractive selection step was performed using pooled serum from individuals seropositive for HIV-1 (*n* = 10). Samples of individuals older than 18 years old, without restriction to ethnicity, social status, or skin color, who were not using corticosteroids were included in the study. Samples of individuals with HTLV-1 who were coinfected with HIV, HBV, or HCV, who had autoimmune diseases, who had an indeterminate diagnosis, or who had a low cut-off value in the enzyme-linked immunosorbent assay (ELISA; Murex HTLV I + II; DiaSorin, Saluggia, Italy) were excluded from the study to minimize potential errors during data analysis and improve the sensitivity and specificity.

### Ethical Aspects

The project was approved by the Human Research Ethics Committee of the Haemotherapy and Hematology Center of Pará (HEMOPA Foundation) under registration number 0011.0.324.000-11. Individuals who agreed to participate in the study signed an informed consent form.

### Phage Display

#### Peptide Selection

Initially, the pool of immunoglobulin G (IgG) in the different groups was linked to magnetic beads (Dynabeads® Protein G; Invitrogen, Waltham, MA, USA) according to the manufacturer's recommendations. Next, the IgG coupled to magnetic microspheres was incubated with a library of random peptides (Ph.D.-C12 Phage Display (PD) Peptide Library Kit; New England Biolabs, Ipswich, MA, USA). The Ph.D.-12 library is a combinatorial library of random 12-mer peptides fused to a minor coat protein (pIII) of M13 phage.

The biopanning procedure was performed as described with some modifications (21). The biopanning process for selection of HTLV-1 peptides included a subtractive step, which consisted of incubating the phage library (1 × 10^11^ phage particles of the PD library) first with IgG from HTLV-seronegative individuals for 30 min at room temperature. Then, the supernatant containing unbound phages was added to IgG of HTLV-2-seropositive individuals with incubation for 30 min at room temperature. The next step consisted of positive selection, in which the final supernatant of the subtractive step was incubated with purified IgG from HTLV-1-positive individuals for 30 min at room temperature. Three biopanning cycles were performed for the selection of HTLV-1 peptides.

The phages bound to the magnetic microspheres of the positive selections were recovered by acid elution and subjected to steps of amplification, titration, supernatant production, DNA extraction, and phage DNA sequencing (21).

#### Phage Amplification

Amplification consisted of phage multiplication in Luria-Bertani (LB) culture medium containing tetracycline (20 mg/mL) and *E. coli* strain ER2738. The medium was incubated under agitation at 37°C until the early-log phase (OD600–0.3). Upon reaching this stage, the bacterial culture was inoculated with the phages and incubated at 37°C overnight under strong agitation. The next day, the material was centrifuged to remove the bacteria, PEG/NaCl was added to the supernatant, the mixture was centrifuged, and the phage pellet was washed with 1× sterile phosphate-buffered saline (PBS).

#### Titration

The titration was performed to determine the amounts of input and output of viral particles during the biopanning cycles. Eluate containing phages from each selection cycle that were not amplified (diluted from 10^−1^ to 10^−5^) and amplified eluate (diluted from 10^−1^ to 10^−12^) were used. *E. coli* ER2738 was cultured in LB medium, to which dilutions containing the phages were added. The samples were plated on LB agar containing tetracycline and isopropyl-beta-d-thiogalactopyranoside/5-bromo-4-chloro-3-indolyl-α-d-galactoside with the addition of Top agar and incubated at 37°C for 24 h.

#### Production of Phage Supernatant

Phage supernatants were produced by amplification of the phages in LB medium (with tetracycline) containing *E. coli* ER2738 in deep-well-plates, which were incubated under agitation in at 37°C for 4–5 h. A different phage clone was added to each well. The phage supernatant was used to back up the clones and extract DNA from the phages.

#### DNA Extraction From Phages

DNA was extracted from the phage supernatant using iodide buffer. The procedure consisted of viral particle lysis, protein precipitation, DNA precipitation, and DNA hydration. The presence of single-stranded DNA was verified by electrophoresis in a 0.8% agarose gel containing HydraGreen Safe DNA Stain 20,000X (Hydragene, Piscataway, NJ, USA) by visual comparison with purified DNA from single-stranded standard M13mp18 tape (New England Biolabs, Ipswich, Massachusetts, USA).

#### Sequencing Reaction

For the sequencing reaction, phage DNA, primer −96 gIII (5'-OH CCC TCA TAG TTA GCG TAA CG-3') (New England Biolabs), and Premix (DYEnamic ET Dye Terminator Cycle Kit; Amersham Biosciences, Amersham, United Kingdom) were used. The reaction was performed in a thermocycler (Kasvi, São José dos Pinhais, PR, Brazil). The sequenced DNA was precipitated and resuspended in dilution buffer and read in the ABI PRISM 3130 Genetic Analyser (Applied Biosystems, Altham, Massachusetts, USA).

#### Data Analysis by Bioinformatics

The DNA sequences obtained after sequencing were analyzed using bioinformatics programs available online. Amino acid sequences were detected *in silico* using the ExPASy Translate Tool 6.0 (http://web.expasy.org/translate/), and the alignment of the selected peptides was generated using the program BioEdit 7.1.5.0 (22). The similarities between the selected peptides and the proteins deposited in GenBank were performed using the Basic Local Alignment Search Tool (BLAST), and within BLAST, the search was performed in the “Protein blast” database using the “swissprot protein sequences (swissprot),” blastp (protein–protein BLAST) algorithm restricted to HTLV (taxid: 11908).

For better characterization of mimetic peptides, B-cell epitope prediction tools were used with the Bepipred Linear Epitope Prediction program (http://tools.iedb.org/bcell) to analyse their antigenicity, hydrophilicity, and accessibility profile. The antigenicity profile was determined using the Kolaskar and Tongaonkar antigenicity scale, a semiempirical method that made use of the physicochemical properties of amino acid residues and their frequency of occurrence in experimentally known epitopes. This scale was developed to predict antigenic determinants in proteins. According to the authors, it can identify antigens with an accuracy of ~75% (23). The hydrophilicity profile was determined through the prediction of Parker hydrophilicity. In this method, a hydrophilic scale was constructed based on the peptide retention times during high-performance liquid chromatography on a reversed-phase column. Parker et al. (24) also found that the hydrophilic surface regions represented are associated with antigenic sites. The accessibility profile was calculated based on the scale of surface accessibility in a product, taking into account the probability of amino acids being exposed on the surface of a protein (25).

### Phage ELISA

Titration curves were generated to determine the most appropriate phage concentration and antibody dilution to be used, to optimize the tests. A microplate (Nunc-Immune Plate MaxiSorp Surface) was sensitized with 10 μg/well anti-M13 monoclonal antibody (GE Healthcare) diluted in 50 mM carbonate buffer, pH 9.6 (1:500), and incubated overnight at 4°C. After washing and blocking the plate, clones diluted in 1× PBS (1:100) were added to the plate and incubated for 1 h at 37°C. The plate was washed, and pools of HTLV-1-positive serum and negative controls were added. After washing, anti-human IgG diluted in 1× PBS/0.1% Tween (1:5000) was added, and the plate was incubated for 1 h at 37°C. Finally, tetramethylbenzidine was added, and the reaction was stopped by the addition of H_2_SO_4_ (2 M). The absorbance values were measured using a reading filter at a wavelength of 450 nm.

A first phage ELISA was performed to evaluate the reactivity of all identified phage clones using the pool of HTLV-1-negative samples (control) and HTLV-1-positive samples. In the ELISA reaction, the samples positive for HTLV-1 were arbitrarily defined as those with an optical density value at least twice as high as the absorbance value of the control samples.

### Statistical Analysis

The calculation of the area under the receiver operating characteristic (ROC) curve (AUC), sensitivity, specificity, and positive likelihood ratio (LR+) was performed using the program GraphPad Prism version 6.0 (GraphPad Prism Software Inc., San Diego, CA, USA). The statistical test used was one-way ANOVA, with post Bonferroni correction test, which is a two-tailed test.

## Results

### Peptides Selected by Phage Display

**Figure 1:**
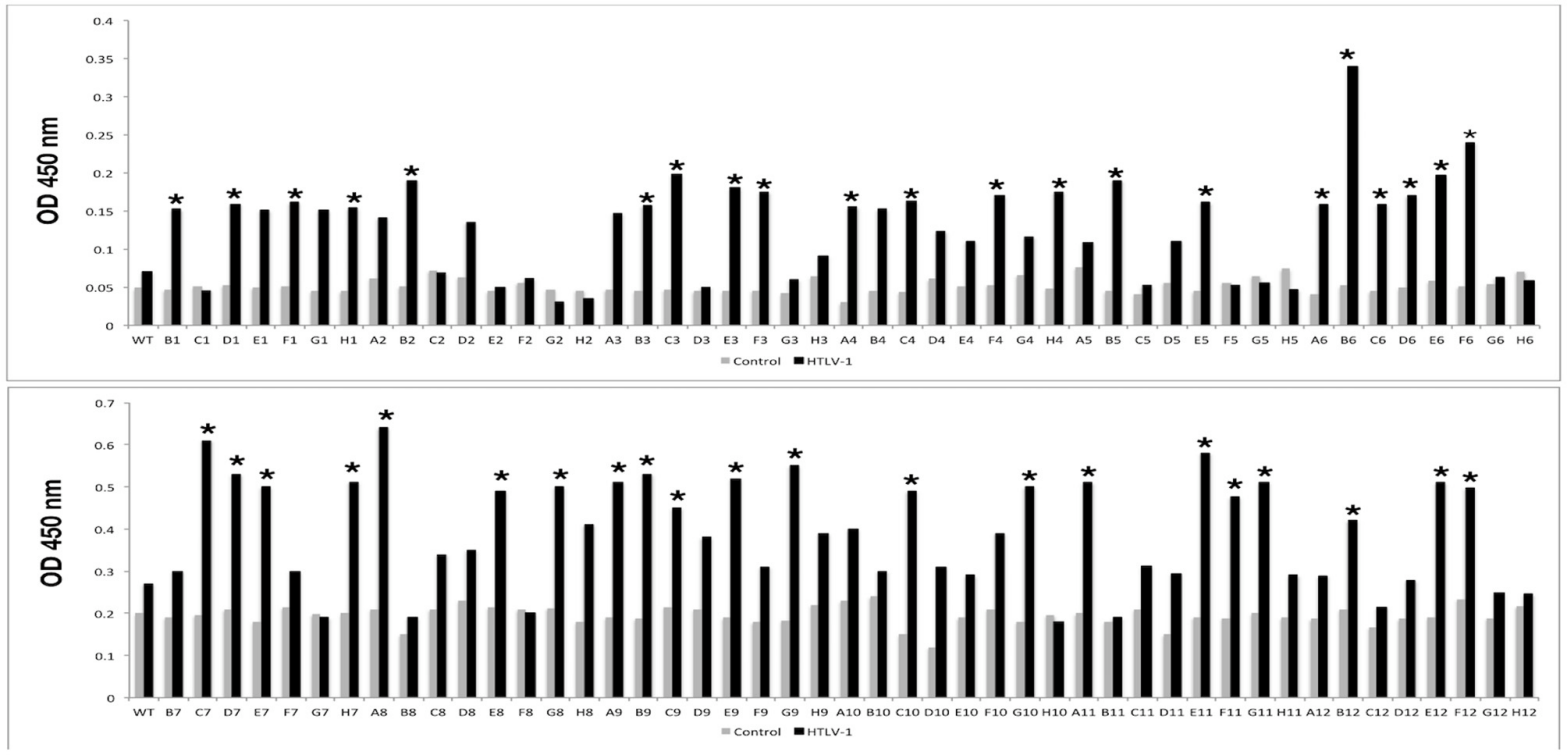
Immunoreactivities of peptides selected by phage display in the Phage-ELISA assay. Peptides that showed significantly higher absorbance when compared to the control (*) were tested against pool serum samples from individuals with HTLV-1 and the control group. WT: wild type phage (phage that does not express any exogenous protein).

After biopanning, a total of 42 selected peptides had different amino acid sequences and were tested for immunoreactivity by phage ELISA (Figure 1).

**Figure 2:**
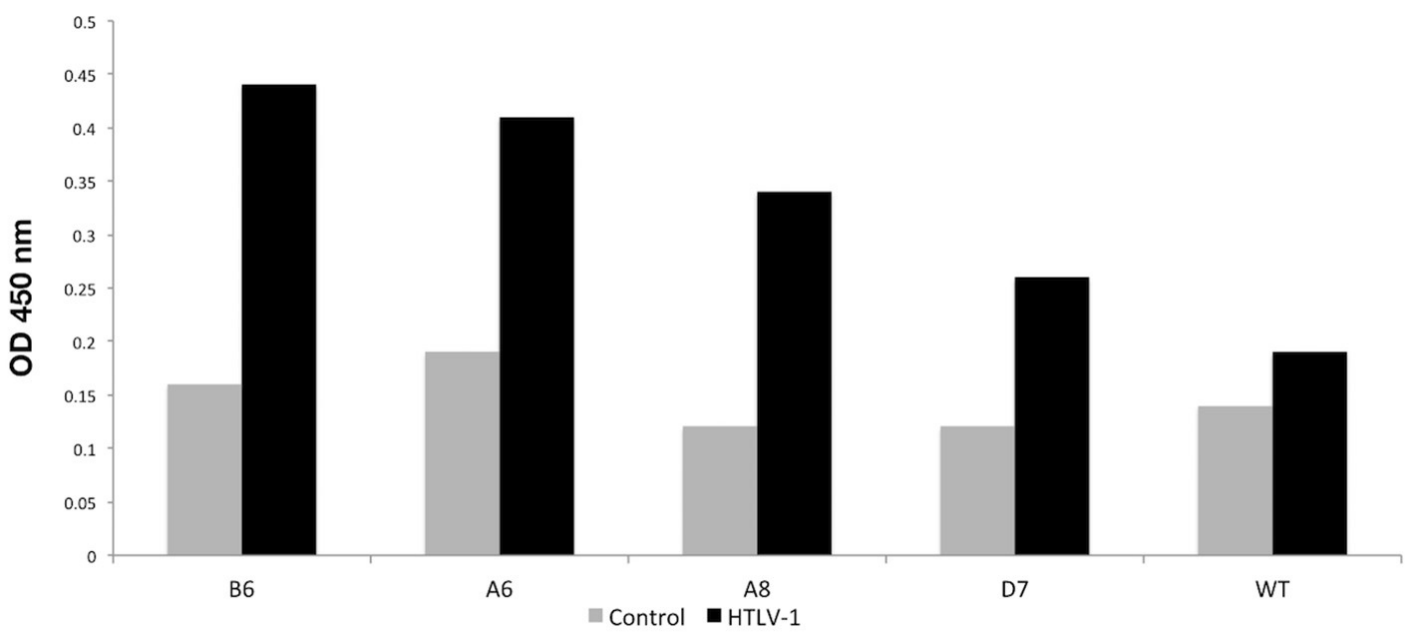
Phage-ELISA assay to test the immunoreactivity of the better-selected peptides against pooled sera from HTLV-1 reagent patients and controls HTLV-1 non-reagent. WT, wild type phage.

After the screening performed in the first Phage ELISA, the phage clones containing the peptides that presented a ratio between the absorbances of the positive and control samples >2 were submitted to a second Phage ELISA. Although most of the peptides could discriminate individuals with HTLV-1 infection from seronegative control individuals, four peptides (A6, A8, B6, and D7) stood out from the rest (Table 1). In the phage ELISA, dilutions of 1:50, 1:100, 1:500, and 1:1000 were used, and the best results were obtained at a dilution of 1:1000 (Figure 2).

**Table 1:** Amino acid sequence of phage clones containing HTLV-1 mimetic peptides after selection by Phage Display.

**Clones**	**Amino acid sequence**
A6	AHWNPFWLATPF
A8	YWVDSSAWVAHK
B6	NNDPLQLRSQRY
D7	KLDVFTKPLVFT

Subsequently, peptide reactivity analysis was performed using individual sera from HTLV-1 seronegative (*n* = 20) and positive individuals (*n* = 10), respectively, for each of the four most reactive clones. For the analysis, the ROC curve, sensitivity, specificity and likelihood ratio (LR) were used (Table 2).

**Table 2:** Clone reactivity when using individual sera from HTLV-1 carriers.

**Peptide**	**Sensitivity**	**Specificity**	**AUC**	***Cut-off***	***P*-value**	**LR+**	**LR-**
A6	72.73 (49.78–89.27%)	72.73 (49.78–89.27%)	0.78	>0.109	0.069	2.7	0.4
A8	68.18 (45.13–86.14%)	72.73 (49.78–89.27%)	0.79	>0.076	0.066	2.3	0.4
B6	77.27 (54.63–92.18%)	77.27 (54.63–92.18%)	0.83	>0.092	<0.001	3.4	0.3
D7	68.18 (45.13–86.14%)	68.18 (45.13–86.14%)	0.69	>0.097	0.299	2.1	0.5

*AUC (Area Under Curve), LR (Likelihood Ratio)*.

**Figure 3:**
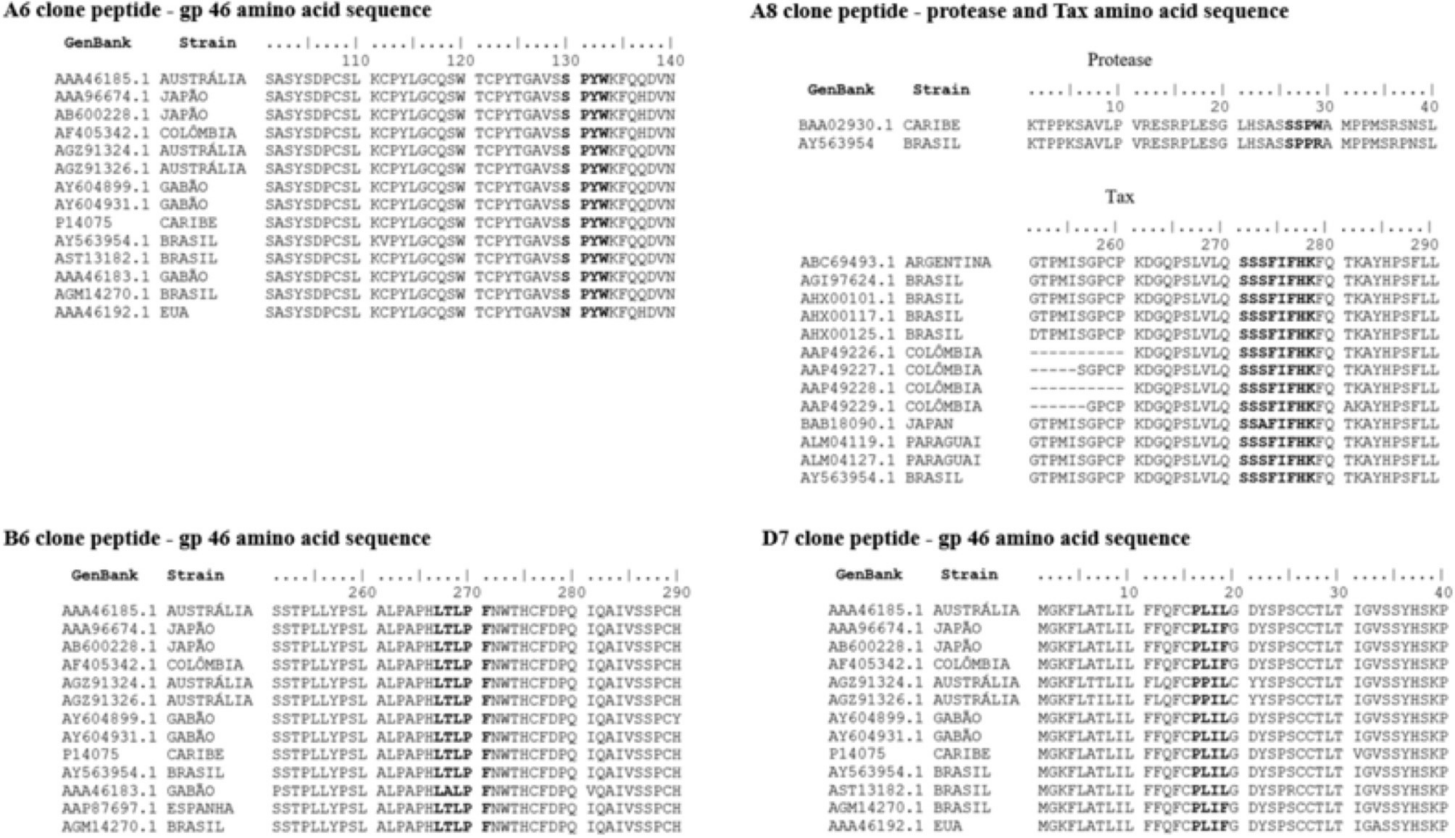
ROC curve of mimetic peptides with AUC values (*Area Under Curve*).

Of the clones tested, only B6 had statistically significant reactivity (*p* < 0.001). It had an AUC equal to 0.83, in addition to having the highest LR+ value (3.4). Thus, this clone could be promising for use in diagnostic immunoassays (Figure 3).

### Alignment of HTLV-1-Mimetic Peptides With HTLV-1 Amino Acid Reference Sequences Available at GenBank

**Figure 4:**
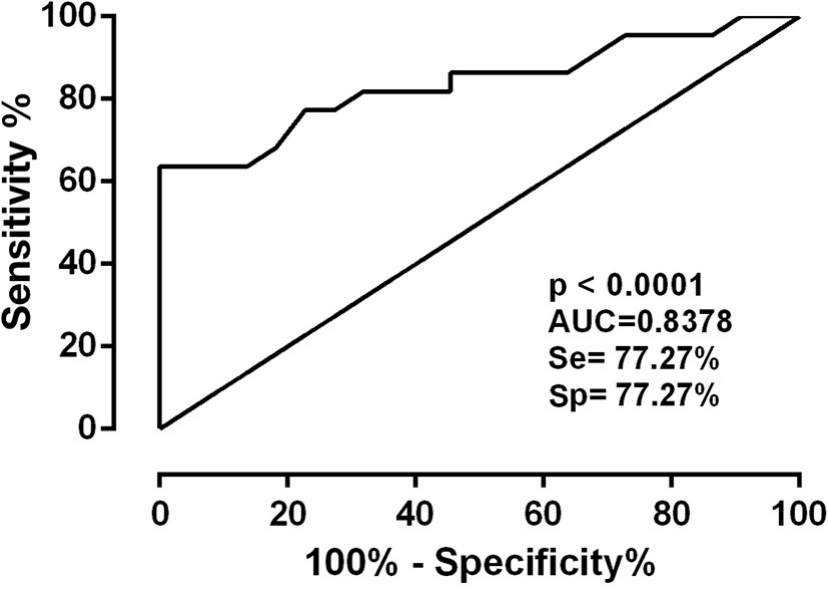
Linear alignment of the amino acid sequences of the four peptides (A6, A8, B6, and D7) with the amino acid sequences of HTLV-1 from various regions of the world available in GenBank.

After linear alignment of the amino acid sequences of the four most reactive peptides in the ELISA with the HTLV-1 amino acid sequences from various regions of the world available in GenBank, we saw that peptides D7, A6, and B6 aligned with sequences of amino acids from different regions of the viral envelope glycoprotein (gp46), while the A8 peptide was aligned with the amino acid sequence of the HTLV-1 protease and Tax protein (Figure 4).

### Antigenicity, Hydrophilicity, and Accessibility Tests

For the antigenicity, hydrophilicity, and accessibility tests, only the Brazilian strain (AY563954.1) was used. In the accessibility profile scale, values above 1 denote regions accessible to antibodies. In the HTLV-1 gp46, the peptide that showed the highest accessibility value was clone A6 (score 2.732), corresponding to the region ranging from amino acids 132–135 (SPYW). Regarding the antigenicity profile, the D7 peptide was the most antigenic (score of 1.176), with a location corresponding to the region between amino acids 13 and 19 (PLIL) of gp46. Hydrophilicity was lowest for peptide D7 (-1.029). In Table 3, we summarize the accessibility, antigenicity, and hydrophilicity values using the Emini, Kolaskar, and Parker scales, respectively, for the clones located in the envelope protein (gp46), protease, and Tax protein.

**Table 3:** Accessibility, antigenicity and hydrophilicity values using the Emini, Kolaskar and Parker scales for the clones located in the envelope protein (gp46), protease and Tax.

**AY563954.1 Position**	**Peptide sequence**	**Accessibility**	**Antigenicity**	**Hydrophilicity**	**Peptide**	**HTLV-1 protein**
16–19	PLIL	0,364	1,176	−1,029	D7	Env
132–135	SPYW	2,732	1,084	0,743	A6	Env
183–186	SQLP	2,366	1,054	2,929	B6	Env
269–273	LTLPF	0,631	1,105	−1,600	A6	Env
28–31	SPPR	1,580	1,022	4,286	A8	Pro
273–278	SFIFHK	0,470	1,068	−0,800	A8	Tax

## Discussion

The immunodiagnostic assays used for peptide detection have evolved with the use of tests such as ELISA and Western blot, although there are false-positive as a consequence of cross-reactions with other aetiological agents (26, 27) and the extensive distribution of different molecular subtypes of HTLV-1 (28) and HTLV-2 (9). The serological assays currently used are based on the detection of antibodies against matrix, nucleocapsid, and envelope proteins, though none of these markers for HTLV-1 have been identified using the phage display technique. The technique has been used in only one study (29), which investigated peptides mimicking HTLV-1 in the cerebrospinal fluid of individuals with HAM. The present study used serum samples from patients with HAM for the bioprospecting of serological markers for the diagnosis of HTLV-1 in symptomatic and asymptomatic individuals, using the phage display technique in the first attempt of this kind in Brazil.

Four peptides characterized as linear mimetopes corresponding to the envelope (gp46), Tax, and protease regions were identified. HTLV-1 envelope glycoproteins, especially gp46, play important roles in the infectious process and are exposed on the surface of viral particles and infectious cells, making them accessible to the immune system (30, 31). Recombinant gp46 proteins from immunogenic regions effectively induce an immune response and the use of a peptide in the outermost region of gp46 (region from amino acid 190–209) was capable to induce antibody responses in 90% of infected individuals (32). In the present study, three mimetopes were mapped to the gp46 region: clones A6, B6, and D7. Clone A6 aligned in two regions (positions 132–135 and 269-273 of the HTLV-1 reference strain AY563954.1). Clones A6, B6, and D7 aligned with gp46 regions corresponding to amino acids 130-133/267-271, 181-184, and 1-4, respectively. These sequences are very close to the regions found by Fujimori et al. (29), which were considered highly homologous to HTLV-1 gp46 in regions 192–199, 237–243, and 255–261.

It was not possible to analyse the 3D structure because the gp46 structure is not available in the RCSB Protein Data Bank, but accessibility, antigenicity, and hydrophilicity of the mimetopes were investigated. Clones A6 and B6 showed values above the cut-off point for all three analyses which is a good indication for using in diagnostic platforms.

Investigations aimed at the development of vaccines have used animal models and *in vitro* assays, but the clinical results have not been satisfactory (33–35). Sequences other than that of HTLV-1 investigated by bioinformatics have predicted epitopes corresponding to Tax and gp62 to be used in the development of vaccines against HTLV-1 (36, 37). The mimetic peptide B6, corresponding to the gp46 sequence, identified from antibodies present in the serum of HTLV-1 infected persons would be a possible candidate for use in future vaccine tests, as it showed satisfactory values of accessibility, antigenicity, and hydrophilicity.

HTLV-1 protease is a nonstructural protein responsible for processing protein precursors into new viral components and is essential for the replication cycle of HTLV-1 (38, 39). Mamoun et al. (40) produced a monomeric form of HTLV-1 protease fused to the maltose-binding protein (MBP-PR) and obtained three monoclonal antibodies capable of recognizing the epitope in different regions of the molecule. Two antibodies were directed against the NH_2_-terminus, a region that contributes to the dimerization interface, and the other was specific to a peptide that lines the substrate-binding region. In the present study, clone A8 showed homology with the protease region, showing accessibility and positive hydrophilicity, though the antigenicity was below the cut-off point.

Clone A8 showed homology with the Tax region. Tax activates viral and cellular gene expression. Its oncogenic potential depends on its ability to alter the expression of cellular genes involved in cell growth and proliferation and their direct interactions with the cycle regulators (41, 42). Although antigenic, clone A8 was not an accessible mimetic and hydrophilic peptide.

The phage ELISA method was used to assess the reactivity of clones against the sera of HTLV-1 infected persons. Screening was initially performed, and the clones with the highest optical density and those with HTLV-1 consensus sequences were selected (clones A6, A8, B6, and D7) for the evaluation of sensitivity and specificity by calculating the ROC. The mimetic peptide (KLDVFTKPLVFT) of clone D7 showed low accessibility and hydrophilicity, which may have hindered the interaction between the peptide and the anti-HTLV-1 antibodies. The best clone in the sensitivity and specificity tests was B6 (NNDPLQLRSQRY), with a sensitivity and specificity of 77.27%, an accuracy of 0.83 (*p* ≤ 0.01), and the highest LR+ value (3.4), all due to its good accessibility, antigenicity, and hydrophilicity.

The study has some limitations such as the sample size, the genetic background of the study population, which included only part of a tri-hybrid Brazilian population, and low HTLV-1 antigenic variation in the study population, since the study did not include samples from areas with other distinct HTLV-1 strains circulating elsewhere. The experiments were performed with peptides fused to the M13 phage. Thus, immunogenicity, antigenicity, positive and negative predictive values, must be further investigated with different conformations of the peptides in order to evaluate their possible use as vaccines for HTLV-1.

In summary, bioprospection of peptides mimicking HTLV-1 using phage display led to the identification of four clones, three related to gp46 (A6, B6, and D7) and one related to protease and Tax (A8). The analysis of accessibility, antigenicity, and hydrophilicity showed that clones A6 and B6 are potentially suitable for diagnostic use and that clone B6 has great potential for use in vaccine tests. The best reactivity, evaluated by phage ELISA, was that of clone B6, which showed good AUC, sensitivity, specificity, and LR. Thus, the successful testing of the B6 clone peptide would be relevant to diagnostic tests on platforms that allow rapid results and to the availability of more affordable testing for the overall population, and it would contribute to the prevention and control of HTLV-1 infection.

## Data Availability Statement

The deposition of the raw data for this manuscript was not possible because of the following reasons: (i) these very small sequences are linked to a bacterial phage (inherent to the phage display methodology), and consequently are not purified; (ii) the sequences are already described as they overlap with gene sequences of glycoprotein 46 (gp46), tax and pro of HTLV-1, which are widely known and fully characterized proteins of the virus, consequently they cannot be registered as “new”; and (iii) the sequences do not reach the size required by the GenBank (50bp). The original contributions presented in the study are included in the article/supplementary material, further inquiries can be directed to the corresponding author/s.

## Ethics Statement

The studies involving human participants were reviewed and approved by Ethics Committee in Research with Human Beings of the Hemotherapy and Hematology Center of Pará (HEMOPA Foundation), under registration number 0011.0.324.000-11. The patients/participants provided their written informed consent to participate in this study.

## Author Contributions

LM, LF, MI, and RI: conceptualization. FS and LS: data curation. FS, LS, AS, and MQ: investigation and methodology. LM, LS, AV, and RI: formal analysis. LM, LF, and LS: writing—original draft. LM, MQ, AV, MI, and RI: writing—review and editing. RI: project administration. All authors contributed to the development of research, read, and approved the final manuscript.

## Funding

This study was supported by Coordenação de Aperfeiçoamento de Pessoal de Nível Superior (CAPES), Pró-Amazônia Program (R.I. Proc. 23038000732/2013-09). LS received fellowships of CAPES, Finance Code 001, to develop the study. AV (#302935/2021-5), RI (#312979/2018-5) and LM (#314209/2021-2) are supported and acknowledge the Research Grants from the Conselho Nacional de Desenvolvimento Científico e Tecnológico (CNPq). Publication of the article was supported by Public Notice PAPQ, PROPESP/FADESP of the Federal University of Pará.

## Conflict of Interest

The authors declare that the research was conducted in the absence of any commercial or financial relationships that could be construed as a potential conflict of interest.

## Publisher's Note

All claims expressed in this article are solely those of the authors and do not necessarily represent those of their affiliated organizations, or those of the publisher, the editors and the reviewers. Any product that may be evaluated in this article, or claim that may be made by its manufacturer, is not guaranteed or endorsed by the publisher.
