# Supplementary material for: Bioprospection and Selection of Peptides by Phage Display as Novel Epitope-Based Diagnostic Probes for Serological Detection of HTLV-1 and Use in Future Vaccines
Source: Front Med (Lausanne). 2022 Jun 9;9:884738. doi: 10.3389/fmed.2022.884738 (PMC9218527; doi:10.3389/fmed.2022.884738)
Supplement: Supplementary file 1 [file Table_1.DOCX]

Bioprospection and selection of peptides by phage display as novel epitope-based diagnostic probes for serological detection of HTLV-1 and use in future vaccines

**Supplementary Material**

Table S1- Characteristics of HTLV-1 patients from the state of Pará, Brazil who participated in this study.

| **Patient** | **Sex** | **Age** | **Infection Time**  **(years)** | **Clinical Manifestation** | **Laboratory Diagnosis** |
| --- | --- | --- | --- | --- | --- |
| 1 | F | 68 | 12 | HAM/TSP* | ELISA/WB** |
| 2 | F | 72 | 9 | Adult T cell lymphoma | ELISA/WB** |
| 3 | M | 28 | 5 | asymptomatic | ELISA/qPCR |
| 4 | F | 55 | 15 | HAM/TSP | ELISA/WB** |
| 5 | M | 37 | 8 | asymptomatic | ELISA/WB** |
| 6 | F | 31 | 6 | asymptomatic | ELISA/qPCR |
| 7 | F | 58 | 13 | HAM/TSP | ELISA/WB** |
| 8 | F | 49 | 9 | asymptomatic | ELISA/WB** |
| 9 | F | 61 | 10 | Rheumatic disease | ELISA/WB** |
| 10 | M | 27 | 4 | asymptomatic | ELISA/qPCR |

* HTLV-1-associated myelopathy/Tropical Spastic Paraparesis; ** Western Blot
